# Vitamin D, Menopausal Health and COVID-19: Critical Appraisal of Current Data

**DOI:** 10.3390/jcm12030916

**Published:** 2023-01-24

**Authors:** Panagiotis Anagnostis, Sarantis Livadas, Dimitrios G. Goulis, Margaret Rees, Irene Lambrinoudaki

**Affiliations:** 1 Unit of Reproductive Endocrinology, 1st Department of Obstetrics and Gynecology, Medical School, Aristotle University of Thessaloniki, 564 29 Thesssaloniki, Greece; 2 Endocrine Unit, Athens Medical Center, 151 25 Athens, Greece; 3 Women’s Centre, John Radcliffe Hospital, Oxford OX3 9DU, UK; 4 Second Department of Obstetrics and Gynecology, Aretaieio Hospital, Medical School, National and Kapodistrian University of Athens, 115 28 Athens, Greece

**Keywords:** vitamin D, menopause, postmenopausal women, vasomotor symptomatology, cardiovascular disease, cancer, COVID-19

## Abstract

Inconsistency exists across studies conducted in postmenopausal women regarding the effect of vitamin D deficiency (VDD) and supplementation on several aspects of menopausal health, such as fractures, vasomotor symptomatology, cardiovascular disease (CVD), cancer and infections, including coronavirus disease 2019 (COVID-19). The aim of this review is to critically summarize the evidence provided by observational studies and randomized controlled trials (RCTs) of vitamin D supplementation in postmenopausal women with VDD. Observational studies have found that VDD is associated with an increased risk of falls and fractures after the menopause. VDD also has a negative effect on menopausal symptomatology. VDD, especially its severe form, is associated with an increased risk of CVD risk factors and CVD events. VDD is associated with increased risk and mortality from several cancer types and risk of infections. The evidence from RCTs regarding the effect of vitamin D supplementation on falls, fractures, menopausal symptoms, cardiovascular disease, cancer and infections is not robust. Thus, skeletal health may benefit only when vitamin D is co-administered with calcium, especially in those ≥70 years old and with severe VDD. There is no evidence of a favorable effect on menopausal symptoms or risk of CVD or cancer, except for a modest reduction in cancer-related mortality. Inconsistency still exists regarding its effect on infection risk, disease severity and mortality due to COVID-19.

## 1. Introduction

Vitamin D plays a fundamental role in calcium and phosphorus metabolism [1]. It is mainly synthesized in the skin under solar ultraviolet B radiation, which transforms 7-dehydrocholesterol into cholecalciferol (D_3_) [1]. After a first hydroxylation in the liver, which results in 25-hydroxyvitamin D [2], a second hydroxylation occurs in the kidneys, where 25(OH)D is transformed into the active metabolite 1,25-dihydroxy-vitamin D (1,25(OH)_2_D) or calcitriol via the enzyme 1α-hydroxylase, which is upregulated by parathyroid hormone (PTH) [1,3]. PTH and vitamin D form a tightly controlled feedback cycle, PTH being a major stimulator of vitamin D synthesis in the kidney while vitamin D exerts negative feedback on PTH secretion. The major function of PTH and major physiological regulator is circulating ionized calcium [4]. Calcitriol exerts its biological actions primarily through the vitamin D receptor (VDR), a nuclear receptor, and is mainly present in the small intestine, kidneys and osteoblasts, although non-genomic actions have also been described [1,3].

Optimal vitamin D status, assessed by serum 25(OH)D concentrations and defined as >20 ng/mL (>50 nmol/L), is essential for the maintenance of musculoskeletal health [3]. Besides its established role in calcium and phosphorus homeostasis, accumulating data provide evidence for the non-skeletal effects of vitamin D [1]. These are mediated through VDRs and 1α-hydroxylase in many tissues, such as immune cells (macrophages, monocytes), cardiomyocytes, vascular smooth muscle cells, and β-cells in the pancreas and reproductive tract tissues [1,5]. Data from experimental and human studies suggest an association between vitamin D deficiency (VDD, defined as 25(OH)D < 20 ng/mL (<50 nmol/L)) [3] and adverse skeletal (osteomalacia, osteoporosis, increased fracture risk) [6] and non-skeletal (cardiovascular disease (CVD), auto-immune diseases, infertility, cancer and infections) [1,5,7] outcomes.

However, it has not been clarified whether the association between VDD and adverse health outcomes indicates causality, since most randomized clinical trials (RCTs) have failed to prove a beneficial effect of vitamin D supplementation on these outcomes [8]. The main reasons for this inconsistency are related to several confounders, such as the short duration of studies, the variation in the populations studied according to ethnicity, skin color, diet, obesity or sun exposure, as well as the low proportion of patients with extremely low 25(OH)D concentrations (<10 ng/mL or <25 nmol/L), in which vitamin D supplementation might have shown some benefit.

This review aims to provide the best evidence from observational studies regarding the association between VDD and skeletal and non-skeletal aspects of menopausal health. Available evidence from interventional RCTs concerning the effect of vitamin D supplementation on these outcomes is summarized.

## 2. Evidence from Observational Studies

The effect of vitamin D on bone metabolism is undisputed, through the genomic activation of VDR on osteoblasts and osteoclasts and regulation of bone mineral metabolism. Thus, VDD results in adverse effects on bone quality and strength, and rises in PTH concentrations will lead to osteoporosis [4]. Postmenopausal women are at high risk of VDD due to endogenous and exogenous factors, such as poor dietary vitamin D intake, diminished exposure to sunlight (mainly in institutionalized women), decreased skin ability to synthesize vitamin D and reduced cholecalciferol absorption by the gut [9]. Moreover, the reduced response of the aging kidneys to PTH leads to decreased concentrations of calcitriol [9]. Finally, the increase in body mass index (BMI) after the menopause is associated with lower 25(OH)D concentrations, which demand a higher replacement dosage [9].

A European study of 8532 women, published in 2007, reported a VDD prevalence of 79.6% in postmenopausal women using the 30 ng/mL (75 nmol/L) threshold [10]. This finding has been confirmed in recent studies, such as that by Valladares et al. (*n* = 21,236), who reported VDD prevalence of 73.6%, 78.6%, 86,1%, 81.5% and 90.4% in Europe, North America, Africa, Middle East and Asia, respectively, by applying the 30 ng/mL (75 nmol/L) threshold [11]. VDD is more common in older women (>80 years): 88.6% and 53.4%, by applying the 30 ng/mL (75 nmol/L) and 20 ng/mL (50 nmol/L) thresholds, respectively [12].

### 2.1. Skeletal Health

A negative association between 25(OH)D concentrations and fracture risk has been established in the population studies. The adjusted relative risk (RR) for fractures in patients with the lowest compared with the highest 25(OH)D concentrations is 1.58 (95% confidence interval (CI) 1.41–1.77), as shown in a meta-analysis of 15 prospective cohort studies (*n* = 51,239 participants; *n* = 3386 cases with hip fractures). Notably, this association was evident only in subjects with 25(OH)D < 60 nmol/L (24 ng/mL), but it was abolished in higher concentrations [13]. On top of a negative association with the risk of hip fractures (RR 1.48, 95% CI 1.29–1.68), a negative association with total fracture risk has also been reported by another meta-analysis (RR 1.25, 95% CI 1.06–1.43) [14].

About 1 in 2 postmenopausal women with osteoporosis or fractures have 25(OH)D concentrations < 15 ng/mL (37.5 nmol/L) [15]. The Women’s Health Initiative (WHI) study, with 400 patients with hip fractures and 400 controls, and a follow-up time of seven years, confirmed these results. Women with the lowest 25(OH)D concentrations (≤47.5 nmol/L (19 ng/mL)) demonstrated higher fracture risk compared with those with the highest concentrations (≥70.7 nmol/L (≥28.3 ng/mL)) (adjusted odds ratio (OR) 1.71, 95% CI 1.05–2.79). This risk increased significantly across 25(OH)D quartiles, independent of falls, physical function, frailty, renal function and estradiol concentrations. Interestingly, each 25 nmol/L (10 ng/mL) decrease in 25(OH)D concentrations was associated with a 33% increase in fracture risk [16].

Finally, the association of VDD with decreased bone mineral density (BMD) in adults is clear at any skeletal site (i.e., hip and spine) where BMD was assessed [17]. This association has also been found in postmenopausal women, independent of ethnic origin [18,19].

In conclusion, an inverse association between 25(OH)D concentrations and the risk of fractures (especially hip fractures), and a positive association with BMD exist. These are mostly evident at low 25(OH)D concentrations (<20 ng/mL), especially in individuals with severe VDD.

### 2.2. Non-Skeletal Health

#### 2.2.1. Menopausal Symptomatology

Few studies have examined the association between VDD and menopausal (vasomotor) symptomatology. A cross-sectional analysis of 530 women (mean age 66.2 ± 6.8 years) from the WHI Calcium and Vitamin D (CaD) trial showed no association between 25(OH)D concentrations and the total number of menopausal symptoms [20]. There was no association between 25(OH)D and individual menopause-related symptoms, such as hot flashes, night sweats, heart racing, sleep disorders, emotional well-being or energy/fatigue, after adjustment for age [20]. In contrast, a cross-sectional analysis of the same subset of postmenopausal women (*n* = 1993) showed an inverse association between joint pain score and 25(OH)D concentrations in unadjusted and adjusted models. The highest mean joint pain scores were evident in the lowest 25(OH)D group (<11.6 ng/mL; <29 nmol/L) [21]. A higher prevalence of VDD in postmenopausal women with hot flashes than in those without was also shown in another cross-sectional study (*n* = 210) after adjustment for age and menopause duration (21.7 vs. 34.2 ng/mL; *p* < 0.001) [22].

Other studies have examined sleep disturbances, depression and sexual function. A meta-analysis (nine studies, 9397 participants) showed an increased risk of sleep disturbances (OR 1.50, 95% CI 1.31–1.72), including poor sleep quality, short sleep duration and sleepiness in individuals with VDD. This effect was independent of study design, sample size, 25(OH)D threshold and geographical location [23]. However, only two studies were conducted exclusively in pre- or postmenopausal women. Another meta-analysis of 11 cross-sectional studies (*n* = 43,137) and five cohort studies (*n* = 12,648) showed an inverse association between 25(OH)D concentrations and the risk of depression, in both sexes, especially for ages ≤ 60 years [24]. Each 10 ng/mL (25 nmol/L) increase in 25(OH)D was associated with a 4% (OR 0.96, 95% CI 0.94–0.99) and 8% (OR 0.92, 95% CI 0.87–0.98) decrease in depression risk (data from cross-sectional and cohort studies, respectively) [24]. Sexual function was inversely associated with 25(OH)D concentrations in a cross-sectional study of 303 postmenopausal women (mean age 53.8 ± 6.2 years) [25].

To conclude, VDD may be associated with some aspects of menopausal symptomatology, although the evidence is not robust and does not derive from studies conducted exclusively in peri- or postmenopausal women.

#### 2.2.2. Cardiovascular Risk Factors

Data from both cross-sectional and cohort studies indicate a negative association between VDD and increased prevalence of CVD risk factors, such as arterial hypertension, dyslipidemia, hyperglycemia and metabolic syndrome, in the general adult population [7].

A meta-analysis published in 2015, including ten prospective and 19 cross-sectional studies, showed a lower risk of arterial hypertension in the general population, with 25(OH)D in the highest compared with the lowest category (prospective studies: RR 0.76 (95% CI 0.63–0.90); cross-sectional studies: RR 0.79 (95% CI 0.73–0.87)). However, this was only found in women ≤ 55 years [26]. This finding was replicated in a cross-sectional analysis of the National Health and Nutrition Examination Surveys (NHANES) 2007–2010, including 2098 premenopausal and 2298 postmenopausal women. In particular, premenopausal women with 25(OH)D < 30 nmol/L (<12 ng/mL) had a 36% lower risk of hypertension compared with those with 25(OH)D ≥ 50 nmol/L (>20 ng/mL) (OR 0.64, 95% CI 0.39–1.02). However, no such association was observed in postmenopausal women [27]. Therefore, current data cannot support a consistent effect of vitamin D status on blood pressure or hypertension in all ages.

Regarding dyslipidemia, data from the NHANES III, the largest study so far, which evaluated the prevalence of CVD risk factors in US adults (*n* = 7186 men and 7902 women), showed a higher prevalence of hypertriglyceridemia (>150 mg/dL) in individuals with 25(OH)D in the first (<21 ng/mL; 52.5 nmol/L) compared with those with 25(OH)D in the fourth quartile (>37 ng/mL; 92.5 nmol/L) (32.9 % vs. 23.8%, respectively; *p* < 0.001). This association (OR 1.47; 95% CI 1.30–1.65) was independent of age, sex and race. However, no association with other lipid parameters was observed [28]. A meta-analysis of 57 cross-sectional (*n* = 210,575) and two cohort studies (*n* = 8494) confirmed the protective effect of highest vs. lowest 25(OH)D concentrations on triglycerides (OR 0.81, 95% CI 0.74–0.89), irrespective of age. This was also the case for low high-density lipoprotein cholesterol (HDL-C) (OR 0.82, 95% CI 0.76–0.89). However, the effect of 25(OH)D on serum triglycerides was marginally non-significant for females (OR 0.86, 95% CI 0.69–1.08), but remained significant for HDL-C [29]. There was no association between 25(OH)D and total (TC) or low-density lipoprotein cholesterol (LDL-C) concentrations [29].

An inverse association between vitamin D status and insulin resistance was found, as assessed by the Homeostatic Model Assessment of Insulin Resistance (HOMA-IR) (r = −0.217; 95% CI −0.161 to −0.272) [30]. Meta-analyses have demonstrated an increased risk of T2DM in patients with VDD compared with those with sufficiency [31,32]. A meta-analysis of prospective studies showed that 25(OH)D concentrations in the highest compared with those in the lowest range were associated with a decreased risk of T2DM and prediabetes by 35% and 51% (RR 0.65 (95% CI 0.55–0.76) and 0.49 (95% CI 0,26–0.93), respectively). Each 10 ng/mL (25 nmol/L) increase in 25(OH)D reduced the risk of T2DM by 12% [31]. However, this benefit was marginally not significant in women (RR 0.83, 95% CI 0.65–1.06) [31].

VDD is associated with an increased risk of metabolic syndrome in postmenopausal women. In a cross-sectional study, metabolic syndrome was detected in 57.8% of women with hypovitaminosis D (including both VDD and vitamin D insufficiency; the latter defined as 25(OH)D 20–30 ng/mL (50–75 nmol/L)) and in 39.8% of those with sufficiency (*p* = 0.003) [33]. In multivariate analysis, 25(OH)D <30 ng/mL (75 nmol/L) increased the risk of metabolic syndrome by 2-fold (OR 1.90, 95% CI 1.26–2.85) [33]. Hypovitaminosis D was positively associated with high triglyceride (OR 1.55, 95% CI 1.13–2.35) and low HDL-C (OR 1.60, 95% CI 1.19–2.40) concentrations compared with VDD [33]. These results were replicated in a cross-sectional study in postmenopausal women (*n* = 616), which showed a higher prevalence of metabolic syndrome in women with VDD compared with those with sufficiency (OR 2.19; 95% CI 1.19–4.01). This association did not change after adjusting for estradiol concentrations. Interestingly, low estradiol concentrations increased the risk of metabolic syndrome in women with VDD [34].

To conclude, VDD is associated with an increased prevalence of CVD risk factors, mainly metabolic syndrome, T2DM and dyslipidemia, particularly high triglyceride and low HDL-C concentrations. Except for VDR in β-pancreatic cells, skeletal muscles and adipose tissue, which mediate insulin secretion and sensitivity, other mechanisms are involved [7]. These include blockade of sterol regulatory element-binding proteins, essential for cholesterol synthesis and uptake [35], reduction of hepatic triglyceride production due to increased calcium uptake by 25(OH)D [36] and PTH suppression, which inhibits lipogenesis and decreases serum triglyceride concentrations [37].

#### 2.2.3. Cardiovascular Disease Events

A growing body of evidence shows an inverse association between 25(OH)D concentrations and CVD prevalence, i.e., coronary heart disease (CHD), stroke and CVD mortality. In particular, patients with severe VDD (25(OH)D <10 ng/mL (<25 nmol/L)) demonstrate a 52% and 72% higher age- and sex-adjusted risk for CHD and myocardial infarction, respectively, compared with individuals with 25(OH)D >50 ng/mL (75 nmol/L) (hazard ratio (HR) 1.52 (95% CI 1.33–1.77) and 1.72 (95% CI 1.40–2.13)) [38]. This is also the case for patients with 25(OH)D 25–49.9 nmol/L (10–19.9 ng/mL), compared with those with 25(OH)D in the highest percentile [38]. The risk of premature death is increased only for the lowest 25(OH)D percentile (<10 ng/mL (<25 nmol/L)) [38]. According to a meta-analysis, low 25(OH)D concentrations are also associated with an increased risk of ischemic stroke (RR 2.45, 95% CI 1.56–3.86) [39].

A meta-analysis, consisting of eight studies with 426,039 patients, confirmed a higher incidence of major adverse CVD events (OR 1.92, 95% CI 1.24–2.98) for patients with VDD, although there was no association with all-cause mortality (OR 1.77, 95% CI 0.75–4.17) [40]. However, VDD, especially the severe form (<10 ng/mL (<25 nmol/L)), was associated with increased CVD mortality in older adults, according to another meta-analysis (RR 1.47, 95% CI 1.15–1.81). Higher 25(OH)D concentrations, but still in the VDD range (10–20 ng/mL (25–50 nmol/L)), were also associated with increased mortality, but to a lesser extent (RR 1.16, 95% CI 1.04–1.27) compared with concentrations >30 ng/mL (>75 nmol/L) [41].

To conclude, VDD, especially the severe form, increases the risk of CVD events (CHD, stroke, mortality), independent of traditional CVD risk factors. Several mechanisms may be proposed for this interplay, such as the presence of 1α-hydroxylase (leading to extrarenal production of calcitriol) and VDRs in endothelial, vascular smooth muscle and immune cells (T-lymphocytes, macrophages), as well as the suppression of PTH secretion, cytokine production (interleukin-2, interleukin-6, interleukin-12, tumor necrosis factor α and β), epidermal growth factor and foam cell production [7]. Vitamin D may downregulate the production of several coagulation factors, such as plasminogen activator inhibitor-1, thrombospondin-1 and thrombomodulin [7]. The contributory role of obesity in the adverse CVD outcomes associated with VDD must also be taken into account [42].

#### 2.2.4. Cancer

VDD has been inversely associated with increased risk of cancer, such as colorectal and lung cancer, and cancer mortality in both sexes, and especially in women. Individuals with 25(OH)D concentrations >25–30 ng/mL (62.5–75 nmol/L) have a lower risk of colorectal and lung cancer by 48% and 84%, respectively, compared with those with VDD [43,44].

Many studies examine the increased risk of breast cancer in women with VDD. In a meta-analysis, women with 25(OH)D concentrations in the highest category (>20 ng/mL or >30 ng/mL) as compared with those with 25(OH)D in the lowest category (<10 ng/mL or <20 ng/mL) present a 15% (data from cohort studies) and 35% (data from case-control studies) lower risk for breast cancer, respectively [45]. However, the protective effect of vitamin D status on breast cancer risk was evident only in premenopausal women (OR 0.67, 95% CI 0.49–0.92) [45], although this was not shown in another meta-analysis [46]. Interestingly, the overall and cancer-specific mortality is increased in postmenopausal women with 25(OH)D concentrations in the lowest (<14 ng/mL (35 nmol/L)) compared with the highest (>22 ng/mL (>55 nmol/L)) tertile (HR 1.52 (95% CI 1.22–1.88) and 1.74 (95% CI 1.23–2.40), respectively), according to a meta-analysis [47]. Another meta-analysis replicated these results, showing reduced overall and disease-specific survival among women with breast cancer and 25(OH)D concentrations in the lowest range compared with those with 25(OH)D at the highest level (mean participant age ranged from 48.8 ± 13.0 to 62.8 ± 5.5 years) [48].

VDD is not associated with an increased risk of ovarian cancer [49,50]. The results for other types of gynecological cancer, such as endometrial and cervical cancers and benign gynecological neoplasms, are conflicting [51,52,53].

In summary, VDD may be associated with increased incidence and mortality of several types of cancer, such as colorectal, lung and breast cancer. The interplay between vitamin D and carcinogenesis is complex, with the most plausible explanations being based on the presence of VDR and 1α-hydroxylase in several tissues, affecting proliferation, apoptosis and angiogenesis [5,54]. The role of VDR polymorphisms should also be considered, although inconsistent data exist concerning breast and ovarian cancer [55,56]. Of note, a Mendelian randomization study provided no genetic evidence for an association between vitamin D and overall cancer outcomes [57].

#### 2.2.5. Infections

VDD may have a negative impact on the immune system by increasing the risk of infections, such as acute respiratory tract infections (ARIs) [58,59], tuberculosis (TB) [60], *Helicobacter pylori* [61], hepatitis B (HBV) [62] and C virus (HCV) [63] and human immunodeficiency virus (HIV) infections [64]. In particular, VDD is associated with a 1.5–2-fold increased risk of community-acquired pneumonia (OR 1.64, 95% CI 1.00–2.67) [58]. Moreover, serum 25(OH)D concentrations are inversely associated with the risk and severity of ARIs (OR 1.83 (95% CI 1.42–2.37) and 2.46 (95% 1.65–3.66), respectively, when comparing the lowest with the highest 25(OH)D category) [59]. According to a prospective cohort study of people with White European ancestry (including 307,601 participants from England, Scotland and Wales, aged 37–73 years), there is a negative association between serum 25(OH)D concentrations and mortality from respiratory diseases [65]. VDD is also associated with a 4-fold increased risk of active TB in subjects with latent TB infection (OR 4.26; 95% CI 2.48–7.30) and tuberculin skin test conversion/TB infection conversion (OR 3.99, 95% CI 1.88, 8.45) [60].

Successful eradication of *Helicobacter pylori* infection is directly correlated to 25(OH)D concentrations. In addition, those with VDD have lower eradication rates than those with more vitamin D [61]. Regarding HBV and HCV infections, an inverse association between 25(OH)D and HBV viral load exists [62], whereas higher sustained virological responses to HCV have been observed in individuals with 25(OH)D >30 ng/mL compared with those with VDD [63]. Furthermore, higher prevalence of VDD has been found in individuals with HIV infection compared with those without HIV (OR 1.52, 95% CI 1.02–2.20) [64].

## 3. Evidence from Interventional Studies

Although the above data favor an inverse association between vitamin D status and adverse health outcomes, they do not necessarily prove causality. The latter could be demonstrated with RCTs by assessing the effect of vitamin D supplementation on these outcomes. However, evidence is characterized by high heterogeneity in terms of the population studied, initial vitamin D status and dosage, calcium co-administration and duration of intervention.

### 3.1. Skeletal Health

Data from RCTs suggest that vitamin D reduces fracture risk only when administered with calcium. According to an umbrella review of meta-analyses of RCTs (follow-up time 1–7 years), vitamin D supplementation, at doses of 400–1600 IU/day, reduced the risk of hip fractures by 16–39% (in eight of 12 meta-analyses) and the risk of any fractures by 5–26% (in seven of 11 meta-analyses), when combined with calcium, at doses of 500–1200 mg/day. This effect was most evident in patients older than 70 years and with baseline 25(OH)D ≤20 ng/mL (≤50 nmol/L) [66]. In a meta-analysis of 54 studies, measures, such as exercise, vision assessment and treatment, environmental assessment and modification, as well as calcium plus vitamin D supplementation (dosage not clearly stated) reduced the risk of injurious falls (OR 0.12, 95% CI 0.03–0.55) [67]. Considering the economic burden of fractures, a European cost–benefit analysis in target populations of adults aged ≥50 years with osteoporosis estimated that 358,453 fractures would be avoided annually if all women over age 50 took calcium and vitamin D supplements. The effect was highest in women >70 years. If this practice was adopted, it would lead to €3.6 billion in cost savings in the EU [68].

However, no anti-fracture benefit was shown in meta-analyses of studies conducted exclusively in community-dwelling individuals (i.e., without osteoporosis or risk factors) [66]. This finding is in accordance with the recently published Vitamin D and Omega-3 Trial (VITAL) study, a two-by-two factorial RCT, which showed no benefit of vitamin D supplementation alone (2000 IU/day) in the general population, including 13,085 postmenopausal women, after a median follow-up of five years. However, 87% of participants did not have VDD at the beginning of the study [69]. In alignment with the VITAL study, a meta-analysis of 11 RCTs for the primary prevention of fractures in adults without VDD, osteoporosis or prior fracture did not disclose any benefit of vitamin D supplementation in doses ranging between 300 IU/day and 100,000 IU/month [70]. A U-shaped effect of vitamin D supplementation on fracture risk should be supported instead, since high vitamin D doses, at monthly (60,000–100,000 IU) or daily intervals (>4000 IU), may increase fracture risk, especially in cases with sufficient vitamin D status [6]. Similarly, an annual oral dose of 500,000 IU, prescribed to 2256 older women (median age 76 years) at high fracture risk, further increased this risk by 25% compared with placebo (adjusted HR 1.25, 96% 0.99–1.58) [71].

Calcium supplementation is safe with regard to CVD risk. Evidence shows increased CVD mortality only in those with daily intake > 1400 mg/day, and not in those with daily consumption of up to 1000 mg/day [72,73]. Given that European postmenopausal women rarely ingest more than 1000 mg/day, their CVD risk is minimal.

The results of vitamin D supplementation and BMD are conflicting. An RCT of 230 postmenopausal women ≤ 75 years with baseline 25(OH)D 14–27 ng/mL (35–67.5 nmol/L) showed a minimal increase (1%) in calcium absorption with twice monthly administration of 50,000 IU vitamin D. However, no change was noticed in BMD at any site compared with controls or those taking a daily vitamin D dose of 800 IU, who did not maintain 25(OH)D concentrations >30 ng/mL (>75 nmol/L) [74]. Nevertheless, vitamin D supplementation may be of benefit in cases with severe VDD (<12 ng/mL (<30 nmol/L)), leading to a significant increase in lumbar or hip BMD [75], with no significant effect at higher 25(OH)D concentrations [76]. In postmenopausal women (aged 50–65 years) with VDD, supplementation with vitamin D 1000 IU/day led to a significant reduction in bone turnover markers, such as serum C-terminal telopeptide of type I collagen and procollagen type 1 N-terminal propeptide, without adverse effects, such as hypercalciuria or hypercalcemia [77].

Another major issue regarding long-term cholecalciferol administration is compliance. Although data are limited, a small study has shown that adult patients prefer solid forms (tablets, capsules) on a monthly schedule rather than liquid forms of cholecalciferol as weekly administration, although 25(OH)D concentrations increased significantly in all participants [78]. Considering the most effective regimen for VDD in postmenopausal women, several approaches based on clinical trials have been proposed. According to a systematic review, daily maintenance doses from 2000 to 4800 IU/day were sufficient to raise 25(OH)D concentrations to an adequate level and preserve it in the long term. On the other hand, daily vitamin D_3_ doses <1000 IU/day have proven inadequate to reach the target 25(OH)D of ≥30 ng/mL (≥75 nmol/L) [79]. Of note, vitamin D dosage should be increased in overweight and obese women to 2.5 and 1.5 times more than in lean subjects [80].

To conclude, healthcare professionals should recommend a combination of calcium plus vitamin D supplementation in postmenopausal women of any age with low serum 25(OH)D concentrations (<20 ng/mL or <50 nmol/L) and osteoporosis or who are at increased risk of fracture. BMI, adherence and compliance to treatment should also be taken into consideration, with regular assessment of 25(OH)D (3–6 monthly intervals) aiming at concentrations >20 ng/mL (50 nmol/L). Maintenance daily doses of 1200–2000 IU (3000–6000 IU in obese patients) in parallel with 1000–1200 mg of calcium, either from dietary sources or supplements, should be encouraged for at least 3–5 years in order to obtain optimal benefits for skeletal health. The evidence of benefit for vitamin D supplementation regarding BMD and fracture in postmenopausal women at low fracture risk remains questionable.

### 3.2. Non-Skeletal Health

#### 3.2.1. Menopausal Symptomatology

In general, few studies have tested the effect of vitamin D supplementation on menopausal symptoms. Data from the WHI CaD trial (*n*= 17,101 postmenopausal women, 50–79 years old) showed no effect of vitamin D 400 IU/day combined with calcium carbonate 1000 mg/day on menopausal symptomatology, either as a whole or as an individual aspect, such as hot flashes, night sweats, sleep disturbances, energy/fatigue or emotional well-being [81]. However, 74% of participants were overweight/obese, and 63% were >60 years. Moreover, baseline 25(OH)D concentrations were not available. This study was the only one included in a meta-analysis published in 2015, showing a beneficial effect of the Mediterranean diet, supplemented with olive oil and soy isoflavone, on cognitive function and memory, in contrast to vitamin D [82]. However, the combination of isoflavones (40 mg/day), calcium (500 mg/day), vitamin D (300 IU/day) and inulin (3 g/day) may improve vasomotor, sexual and physical domains after 12 months of treatment, according to a recent RCT (*n* = 50 postmenopausal women; baseline 25(OH)D concentrations: 30.5 ± 5.2 ng/mL (76.2 ± 13 nmol/L)) [83].

With regard to depression, a meta-analysis of nine RCTs (three in postmenopausal women) showed no effect on depression scores with cholecalciferol at a dose of 500,000 IU/year or 400 IU/day, combined with calcium and anti-depressants or calcitriol 0.25 μg/day. Of note, these studies included individuals with a low depression prevalence and vitamin D sufficiency at baseline [84]. However, a meta-analysis of 15 RCTs did show some effect with daily doses from 400 to 18,400 IU. Nonetheless, of six RCTs in postmenopausal women, five did not show any effect (three used doses as described above, and two 400–800 IU/day, either alone or with calcium) [85]. Only one RCT showed a beneficial effect of vitamin D_3_ 18,400 IU/day (combined with lycopene, astaxanthin and citrus bioflavonoid) on the total menopausal symptomatology score (45% reduction), as well as on hot flashes, depression, anxiety, incontinence and joint pain [86]. These findings were replicated in more recent RCTs, with low baseline vitamin D concentrations (mean 25(OH) 11.6 ± 2.6 nmol/L (4.6 ± 1.0 ng/mL), which increased to 77.9 ± 40. 6 nmol/L (31.2 ± 16.2 ng/mL) after 12 weeks) [87]. Regarding sleep quality, there is no evidence from RCTs for any beneficial effect of vitamin D supplementation, without any difference according to baseline vitamin D status [88,89].

Vitamin D supplementation has a potential benefit regarding postmenopausal genitourinary syndrome. A systematic review that included six studies (two RCTs and four quasi-randomized trials) showed an improvement in vaginal atrophy over placebo, when vitamin D was administered as a vaginal suppository at daily doses of 1000 IU or orally at 60,000 IU weekly doses (data from the two RCTs). However, no effect was reported for oral doses of 200–1000 IU/day combined with 500 mg calcium/day (data from the four quasi-randomized trials) [90]. This favorable effect on vulvovaginal atrophy (assessed by vaginal maturation index, vaginal pH and the visual analog scale) symptoms was shown in an RCT after 12 weeks with oral ergocalciferol, at a dose of 40,000 IU/week, (*n* = 80) (baseline 25(OH)D concentrations in the vitamin D and placebo groups: 25.0 ± 8.3 and 23.3 ± 7.5 ng/mL, respectively; 25(OH)D at the end of the study: 39.9 ± 13.5 and 22.6 ± 7.0 ng/mL, respectively) [91].

#### 3.2.2. Cardiovascular Risk Factors

Vitamin D supplementation may have a modestly beneficial effect on the postmenopausal lipid profile. According to a meta-analysis of seven RCTs, vitamin D (300–4000 IU/day) may decrease TG concentrations (weighted mean difference (WMD) −3.55 mg/dL, 95% CI −5.34 to −1.76) and increase HDL-C, only when the treatment duration was ˂26 weeks (WMD 2.67 mg/dL, 95% CI 0.66–4.68), as well as TC concentrations (WMD 6.56 mg/dL, 95% CI 0.78–12.35). However, a vitamin D dose of ˃400 IU/day may slightly decrease LDL-C (WMD −1.89 mg/dL, 95% CI −2.47 to −1.31) [92]. Although to a lesser extent, these alterations in TG and HDL-C concentrations of postmenopausal women were also shown in another meta-analysis, but without any effect on LDL-C and TC (vitamin D dosage: 300–4000 IU/day) [93]. No data on baseline 25(OH)D concentrations were available from these two meta-analyses. Furthermore, vitamin D supplementation has no effect on systolic or diastolic BP in the general population, according to a meta-analysis of 27 RCTs. Mean or median baseline 25(OH)D concentrations varied from 25.6 nmol/L to 78 nmol/L (10.2 ng/mL to 31.2 ng/mL). Eleven RCTs were conducted in populations with VDD or vitamin D insufficiency. No difference according to age, baseline vitamin D status or vitamin D dose (i.e., ≤800 or >800 IU/day) was observed [94].

Vitamin D supplementation may also improve metabolic syndrome profile in postmenopausal women. According to an RCT (*n* = 160 postmenopausal women aged 50–65 years old), vitamin D_3_ 1000 IU/day (*n* = 80) reduced triglyceride concentrations and improved HOMA-IR compared with placebo (*n* = 80) after nine months of treatment. No effect on the other parameters, such as TC, LDL-C, HDL-C and fasting plasma glucose, was observed (25(OH)D in vitamin D and placebo groups at baseline: 15.0 ± 7.5 vs. 16.9 ± 6.7 ng/mL, respectively; at the end of the study: 27.5 ± 10.4 vs. 13.8 ± 5.9 ng/mL, respectively) [95].

Vitamin D supplementation does not affect the incidence of T2DM in the general population, according to a recent meta-analysis (nine RCTs, mean 25(OH)D concentrations >18 ng/mL (45 nmol/L) in five; 20–25% with severe VDD) [96]. However, it may delay the development of T2DM in patients with prediabetes by 11% (RR 0.89, 95% CI 0.80–0.99), according to a meta-analysis (eight RCTs, mean 25(OH)D ranging from 30.7 nmol/L (12.3 ng/mL) to 69.2 nmol/L (28.8 ng/mL); four studies with 25(OH)D <50 nmol/L (20 ng/mL)). No difference according to baseline 25(OH)D (≥50 or <50 nmol/L) was observed [97]. Of note, both meta-analyses found a favorable effect only in patients with BMI <30 kg/m^2^ and with vitamin D daily doses ≥2000 IU [97] or >1000 IU, and in individuals ≥60 years [96].

#### 3.2.3. Cardiovascular Disease Events

Several studies have assessed the potential benefit of vitamin D supplementation regarding CVD events, but with high heterogeneity regarding the dosage, formulation, duration of treatment and population studied. In a meta-analysis of 21 RCTs (*n* = 83,291; mean age 65.8 ± 8.4 years; 74.4% women), vitamin D supplementation (from 400 to 100,000 IU/month) did not reduce the risk of total CVD events, myocardial infarction, stroke, CVD or all-cause mortality (mean baseline 25(OH)D concentrations ranged from 7.3 ng/mL (18.2 nmol/L) to 53 ng/mL (132.5 nmol/L); >20 ng/mL (>50 nmol/L) in the vast majority of studies) [98]. These findings were not modified by sex, baseline 25(OH)D concentrations, vitamin D dosage, formulation and calcium co-administration [98]. A null effect of vitamin D on CVD was also shown in a Cochrane meta-analysis (ten RCTs, 47,267 participants; vitamin D dosage ranging from 400 IU/day to 100,000 IU/month or 150,000 IU/3 months) [99] and the updated evidence report and systematic review for the US Preventive Services Task Force (seven RCTs, 74,925 participants; vitamin D dosage ranging from 400 IU/day to 100,000 IU/month or 150,000 IU/3 months) [100].

#### 3.2.4. Cancer

The effects of vitamin D supplementation on cancer incidence and related mortality are conflicting. Vitamin D supplementation (at doses of 400 IU/day to 100,000 IU/4 months, mostly 2000 IU/day) may reduce the incidence (OR 0.87, 95% CI 0.82–0.92) and extend survival in patients with colorectal cancer (proportion of patients with VDD across studies: 13–99%) [101,102].

Vitamin D (1200 IU/day) improved survival only in the subgroup of patients with early-stage lung adenocarcinoma and those with 25(OH)D <20 ng/mL (<50 nmol/L) [2]. No effect of vitamin D supplementation (400 IU/day) plus calcium (1000 mg/day) on lung cancer incidence was shown in postmenopausal women enrolled in the WHI trial (no data on baseline vitamin D status were available) [103]. Vitamin D supplementation (400 IU/day to 100,000 IU/month), either alone or with calcium, has no effect on the risk of breast cancer (mean baseline 25(OH)D concentrations ranging from 12.8 ng/mL (32 nmol/L) to 32.8 ng/mL (82 nmol/L); half of the studies with VDD) [104,105]. This is also the case for ovarian cancer [49]. It must be mentioned that, regarding ovarian cancer, data were derived from case-control and cohort studies, which reported the total vitamin D intake, mainly from dietary sources. No association between serum 25(OH)D concentrations and ovarian cancer risk was reported [49]. Of note, the updated systematic review by the US Preventive Services Task Force did not show any beneficial effect of vitamin D on cancer incidence (OR, 0.98, 95% CI 0.92–1.03) (vitamin D dosage ranging from 400 IU/day to 100,000 IU/month or 150,000 IU/3 months) [100].

A Cochrane meta-analysis of patients with malignancies reported a slight decrease in all-cause mortality (RR 0.88, 95% CI 0.78–0.98). However, this effect was not evident in studies that included only female populations (RR 0.93, 95% CI 0.83–1.03) (vitamin D dosage ranging from 400 IU/day to 100,000 IU/month or 150,000 IU/3 months). No difference according to vitamin D status at baseline was observed [99]. An updated meta-analysis of 50 trials confirmed this reduction in cancer-related mortality (RR 0.85, 95% CI 0.74–0.97) (no difference was observed according to vitamin D dose, <2000 or ≥2000 IU/day). Baseline 25(OH)D concentrations were <10 ng/mL (<25 nmol/L) only in four trials and 10–20 ng/mL (25–50 nmol/L) in 21 [106].

#### 3.2.5. Infections

Heterogeneity among studies exists concerning the effect of vitamin D supplementation on the risk of ARIs. A meta-analysis of 43 studies (*n* = 48,488) showed a modest benefit (OR 0.92, 95% CI 0.86–0.99), regardless of baseline 25(OH)D concentrations. In subgroup analysis, this effect was evident only with 400–1000 IU/day for a duration of ≤12 months and only for participants aged <16 years at enrolment [107]. Another recent meta-analysis of 30 RCTs (*n* = 30,263) failed to show such a benefit (RR 0.96, 95% CI 0.91–1.01) (vitamin D dosage across studies: 400 IU/day to 100,000 IU/month). In subgroup analysis, daily (RR 0.83, 95% CI 0.73–0.95) and short-term vitamin D supplementation (RR 0.83, 95% CI 0.71–0.97) was efficacious, although this effect lost significance when the analysis was confined to high-quality studies [108]. Inconclusive results emerged from a systematic review of meta-analyses and RCTs regarding the effect of vitamin D supplementation on ARIs. However, individuals that could benefit were those with VDD at baseline or high-risk conditions [109].

## 4. Vitamin D and COVID-2019

### 4.1. Data from Epidemiological Studies

Numerous studies and meta-analyses have been conducted to assess the association between vitamin D status and the severity of coronavirus disease 2019 (COVID-19) infection. A meta-analysis of 17 observational studies (*n* = 2756, age range 43–86 years), published in 2022, showed higher mortality (OR 2.47, 95% CI 1.50–4.05) and hospitalization rates (OR 2.18, 95% CI 1.48–3.21) for subjects with VDD compared with those with sufficiency. Subgroup analysis according to VDD definition, geographical location and latitude did not change these results [110]. The positive association between VDD and severity or mortality of the disease was replicated by another meta-analysis of 26 observational studies (*n* = 8176, age range 54–62 years), published in 2022, although there was no effect on the chance of COVID-19 infection [111]. Another meta-analysis of 16 observational studies (*n* = 386,631 patients, age range 8–76 years) comparing vitamin D status in COVID-19 positive and negative patients found lower 25(OH)D concentrations in the former group (mean difference (MD) −1.70 ng/mL; 95% CI −2.74 to −0.66; *p* = 0.001). This difference was more evident in women than in men. However, the results varied according to study design, since no difference was found between groups in cohort studies (MD −0.39 ng/mL, 95% CI −1.62 to 0.84; *p* = 0.538), in contrast to case-control studies (−4.04, 95% CI −5.98 to −2.10; *p* < 0.001) [111]. Furthermore, a large cohort study (*n* = 307,512 participants, 54.9% females, 55.9% >70 years), not included in these meta-analyses, which used data from the UK Biobank, found no evidence that VDD or vitamin D insufficiency is associated with either hospitalization or mortality due to COVID-19, despite a modest increase in the risk of infection (HR 1.14, 95% CI 1.01–1.30) [112].

To conclude, there is evidence—although not robust—for an increased risk of COVID-19 infection, as well as higher hospitalization and mortality rates in patients with VDD compared with those with sufficient vitamin D status.

### 4.2. Data from Interventional Studies

Interpretation of interventional studies is limited by the high heterogeneity in study design, different participant populations, as well as duration and dosage of supplementation. No firm conclusions can be drawn due to the paucity of data from RCTs. According to the most recent meta-analyses, vitamin D supplementation at a bolus dose of 50,000–200,000 IU, followed by maintenance dose ranging from 800 IU/day to 10,000 IU/week or 50,000 IU/month, may decrease the risk of admission to the intensive care unit by 54–65%, although it has no effect on the risk of COVID-19 infection [113,114]. Results regarding COVID-19-associated mortality and risk of infection are conflicting [113,114,115].

Another meta-analysis (eight RCTs, *n* = 657) showed no effect of vitamin D supplementation on mortality rates, length of hospitalization, intensive care unit (ICU) admission or mechanical ventilation due to COVID-19 infection [116]. The vast majority of the studies included were of small size (16–50 participants; one study included 119), four of which were conducted in VDD populations (defined either as 25(OH)D <20 ng/mL or <30 ng/mL), with high heterogeneity in terms of dosage and duration (daily dose of 125, 250, 500 or 1500 μg for 14 days (*n* = 5 studies); loading dose of 5000 μg (*n* = 2 studies); loading dose of 532 μg, followed by 266 μg on days 3, 7, 14, 21 and 28 (*n* = 1 study)) [116]. The ongoing Vitamin D for COVID-19 (VIVID) trial, which is a double-blinded RCT, designed to assess the effect of vitamin D supplementation (loading dose, then 3200 IU/day for four weeks) or placebo in a 1:1 ratio, on hospitalization rates and/or mortality due to COVID-19, as the primary outcome, should provide further evidence. Secondary outcomes include difference in symptom severity scores and changes in the infection status between groups [117].

Thus, although vitamin D supplementation may modestly decrease the severity of COVID-19 infection, the high heterogeneity among studies necessitates the conduction of new RCTs in order to determine the optimal vitamin D dosage and duration in order to prevent adverse COVID-19-related outcomes. Furthermore, non-pharmacological options, such as regular exercise, may also be considered. This low-cost intervention may exert a favorable effect on the immune system and can be carefully implemented, since it may protect against ARIs and COVID-19 infections and increase post-vaccination immune responses, especially in the elderly population [118].

## 5. Critical Appraisal of Data

The discrepancy between RCTs and observational studies may be attributed to several reasons, such as the short duration (mainly of studies assessing CVD and cancer risk), the inclusion of patients in generally good health and low CVD risk, and the low proportion of those with severe VDD, in which vitamin D could show some benefit. Moreover, the association between VDD and adverse health outcomes may have been driven by confounding or reverse causality. In particular, chronic conditions or other factors, such as physical inactivity due to illness or obesity, medications or bariatric surgery, may lead to VDD (i.e., due to reduced sun exposure) rather than vice versa. Furthermore, the duration of VDD and the effect of potential fluctuations in 25(OH)D and PTH concentrations on these outcomes should also be considered. Finally, patients with VDD who could benefit most from vitamin D supplementation are those with severe VDD (<10 ng/mL or <25 nmol/L). However, this subpopulation was under-represented in most RCTs (10%). Moreover, baseline 25(OH)D concentrations were close to the lower limit of normal (>16 ng/mL (>40 nmol/L) in 70% of RCTs), which could compromise any potentially favorable effect of vitamin D supplementation [119].

In general, despite the inconsistency across international guidelines, most propose 25(OH)D concentrations ≥20 ng/mL (≥50 nmol/L) as essential for the maintenance of optimal skeletal health. An international consensus on the definition of adequate vitamin D intake and the exact effect of supplementation in older people is still lacking. Most societies, such as the Institute of Medicine, the European Calcified Tissue Society and the International Osteoporosis Foundation, recommend daily vitamin D doses of 400–800 IU, as a general rule, although evidence of the anti-fracture efficacy of this policy is lacking [120].

Modest anti-fracture and anti-fall efficacy may be observed with vitamin D supplementation at doses of 800–2000 IU/day, only in combination with calcium (1000–1200 mg/day), especially in elderly populations with severe VDD (25(OH)D < 10–12 ng/mL (<25–30 nmol/L)) [6]. Notably, high compliance and a duration of >3 years are essential for such a benefit [6]. There is no evidence for vitamin D supplementation in those with 25(OH)D concentrations >20 ng/mL and at low risk for fracture.

Higher 25(OH)D concentrations (>30 ng/mL (>75 nmol/L)) may be needed for non-skeletal effects, although the evidence from RCTs is weak. Vitamin D supplementation may benefit cases of vulvovaginal atrophy, prediabetes and COVID-19. In the latter two, optimal vitamin D intake may reduce the risk of progression to T2DM and severe (requiring admission to ICU) COVID-19 infection, respectively. Vitamin D supplementation is not recommended for managing menopausal symptomatology (i.e., hot flashes, night sweats, depression, sleep disorders, sexual dysfunction), nor for CVD or cancer prevention.

With regard to dosage, all postmenopausal women with severe VDD should be initially treated with 50,000 IU/week or 6000 IU/day of vitamin D for eight weeks to achieve 25(OH)D concentrations >20 ng/mL (ideally >30 ng/mL), followed by maintenance therapy of 1500–2000 IU/day. In obese patients, the initial and maintenance doses should be 2–3 times higher [3]. For those with 25(OH)D 12–20 ng/mL, the evidence for optimizing musculoskeletal health and reducing fracture risk exists only for older postmenopausal women (>65 years), with doses of 1000–2000 ΙU/day in combination with calcium (1000 mg/day, either as a supplement or from dietary sources) [6,121].

Inconsistency still exists concerning the 25(OH)D threshold for defining vitamin D sufficiency (>20 ng/mL or >30 ng/mL) [121].

## 6. Conclusions

In conclusion, VDD, mainly its severe form, compromises menopausal skeletal health, since it is associated with low BMD and increased risk of fractures. It may also negatively affect some aspects of menopausal symptomatology, although the evidence is not robust. Moreover, VDD may increase CVD risk, the incidence and mortality of several types of cancer and risk of infections, such as ARIs, including COVID-19. Concerning vitamin D supplementation, heterogeneity exists among studies in terms of baseline 25(OH)D concentrations, duration of VDD, population characteristics (e.g., ethnicity, obesity status, sun exposure), vitamin D dosage, population studied, calcium co-administration and duration of intervention. In any case, there is an exigent need for future RCTs to determine the optimal 25(OH)D threshold required for vitamin D supplementation, its target range and the optimal vitamin D dosage and treatment duration to get the maximum skeletal and non-skeletal benefit.

## Data Availability

Data is contained within the article.
